# A Tool for Classifying Individuals with Chronic Back Pain: Using Multivariate Pattern Analysis with Functional Magnetic Resonance Imaging Data

**DOI:** 10.1371/journal.pone.0098007

**Published:** 2014-06-06

**Authors:** Daniel Callan, Lloyd Mills, Connie Nott, Robert England, Shaun England

**Affiliations:** 1 Center for Information and Neural Networks, National Institute of Information and Communications Technology, Osaka University, Osaka, Japan; 2 Chronic Pain Diagnostics, Roseville, California, United States of America; University of Maryland, College Park, United States of America

## Abstract

Chronic pain is one of the most prevalent health problems in the world today, yet neurological markers, critical to diagnosis of chronic pain, are still largely unknown. The ability to objectively identify individuals with chronic pain using functional magnetic resonance imaging (fMRI) data is important for the advancement of diagnosis, treatment, and theoretical knowledge of brain processes associated with chronic pain. The purpose of our research is to investigate specific neurological markers that could be used to diagnose individuals experiencing chronic pain by using multivariate pattern analysis with fMRI data. We hypothesize that individuals with chronic pain have different patterns of brain activity in response to induced pain. This pattern can be used to classify the presence or absence of chronic pain. The fMRI experiment consisted of alternating 14 seconds of painful electric stimulation (applied to the lower back) with 14 seconds of rest. We analyzed contrast fMRI images in stimulation versus rest in pain-related brain regions to distinguish between the groups of participants: 1) chronic pain and 2) normal controls. We employed supervised machine learning techniques, specifically sparse logistic regression, to train a classifier based on these contrast images using a leave-one-out cross-validation procedure. We correctly classified 92.3% of the chronic pain group (N = 13) and 92.3% of the normal control group (N = 13) by recognizing multivariate patterns of activity in the somatosensory and inferior parietal cortex. This technique demonstrates that differences in the pattern of brain activity to induced pain can be used as a neurological marker to distinguish between individuals with and without chronic pain. Medical, legal and business professionals have recognized the importance of this research topic and of developing objective measures of chronic pain. This method of data analysis was very successful in correctly classifying each of the two groups.

## Introduction

Chronic pain, defined as pain that persists for an extended time after the injury has completed the healing phase [4]–[5], is one of the most prevalent health problems in developed countries [1]–[3]. It is estimated that chronic pain affects more than 100 million Americans [1], [5]–[7]. The increased medical expenses, lost income and lowered productivity make chronic pain one of the most costly health problems in the world.

Despite the importance, interest, and expense associated with chronic pain, there is still no widely accepted objective measure of chronic pain. Diagnosis of chronic pain is based primarily on the subjective reports of the individual [8], [9], or subjective reports by care providers. However, cognitive, communicative, and psychological impairments, as well as deception [9]–[15], preclude the reliable use of self-reports in certain situations. While objective measures of pain may not replace subjective pain ratings as argued by Robinson et al., [8] and Sullivan et al., [16], it is likely that brain based objective measures of pain may augment and facilitate diagnosis, treatment, and promote better understanding of the underlying cause of different types of pain [15], [17]–[20]. For example, while a doctor may identify the presence of a fever by asking the patient if they feel hot or cold, an objective measure of temperature taken by a thermometer greatly improves the diagnosis.

Recently, several studies have focused on assessing the presence and sensitivity of acute pain by applying multivariate pattern analysis to brain imaging data [9], [13], [21]–[26] (See [27] for a comprehensive review of applying machine learning algorithms to pain neuroimaging). Many of these studies were successful at classifying the presence or absence of pain in normal healthy individuals using functional magnetic resonance imaging (fMRI) brain activity data. Depending on the nature of the decoding task and training/testing method, classification performance was as follows: Thermal pain tolerance versus temperature sensation: 91% using Gaussian process classification [21]; 86% using relevance vector machines [21]; 91% and 86% respectively using support vector machines [21], [13]; 95% using least absolute shrinkage and selection operator regularized principal components regression [9]. Thermal pain detection versus thermal pain tolerance: 71% using Gaussian process classification; [21]; 69% using relevance vector machines [21], 68% using support vector machines [21]. Thermal pain detection versus temperature sensation: 71% using Gaussian process classification [21]; 68% using relevance vector machines [21]; 91% using support vector machines [21]; 61% using laser stimulated near-threshold painful from non-painful trials [24]. The study by Wager et al., [9] in particular defines a ‘neural pain signature’ that may be a universal signature for pain composed of weighted activity from many brain regions that may be generalized across subjects and is sensitive to different types of pain. It is unclear, however, whether the ‘neural pain signature’ derived from acute pain will generalize to chronic pain [28].

There is evidence supporting the position that individuals with chronic pain may have a functional reorganization in brain regions associated with pain compared to individuals without chronic pain [3], [29]–[31]. This functional reorganization may be the cause of their symptoms in the absence of any physical injury to the body. This reorganization may manifest itself in the form of differences in intrinsic brain connectivity reflected in resting state networks that have been shown to differ in individuals with chronic pain [31]. Cortical reorganization in individuals with chronic pain has also been shown to manifest itself in regional differences in grey matter density [5], [30]–[35]. In particular the study by Ung et al. [35] utilizes multivariate pattern analysis to successfully classify the presence or absence of chronic pain using morphological differences in the anatomical magnetic resonance images (MRIs) of 47 patients and 47 normal controls with an accuracy level of 76%.

We hypothesize, based on long-term reorganization in brain processes associated with chronic pain, that individuals suffering from chronic pain do have different patterns of brain activity that can be recorded by fMRI. In this experiment we record alternating periods of painful stimulation each followed by a period of no stimulation using fMRI brain scanning. Since there is known variability in subjective pain sensation in different individuals [5] we chose to investigate brain activity in response to each individual’s subjective maximum tolerable level in this experiment. Multivariate pattern analysis, in this case, sparse logistic regression (SLR) [36], is predicted to be able to recognize differences of brain activity between individuals with chronic pain and normal controls in response to induced pain. A leave-one-out cross-validation procedure is used to train and test the SLR classifiers based on differential activity in contrast images of induced pain relative to rest in specific brain regions thought to be involved with pain processing (Including: primary somatosensory cortex, secondary somatosensory cortex, inferior parietal cortex, insula, and anterior cingulate cortex [3]). A classifier is defined as a set of variables (in our case, weights of the extracted features of the SLR) that sorts the data samples into different categories. Using a leave-one-out cross-validation procedure the total number of classifiers is equal to the number of participants to be classified. Each classifier is trained on data for all participants except the one upon which it is tested.

Our study extends the use of fMRI data and multivariate pattern analysis techniques to classify individuals with and without chronic pain. Many applications of multivariate techniques to fMRI data have been designed to classify types of stimulus, conditions or trials within a single individual using the blood oxygenation level dependent (BOLD) activity from several scans as input from the same individual [13], [21], [24]. Our goal differs from these studies in that it seeks to demonstrate generalized diagnostic performance for identifying presence or absence of chronic pain for individuals not used during training of the classifier.

## Methods

### Ethics Statement

All participants were informed of the experimental procedures to be used. All procedures were approved by the New England Institutional Review Board (NEIRB) in accordance with the principles expressed in the Declaration of Helsinki. All participants gave written informed consent prior to the experiment. Participants were paid for taking part in the experiment.

### Subjects

Twenty-six screened individuals participated in this study. Thirteen of the participants had chronic pain (chronic pain group) and thirteen of the participants had no chronic pain (normal, control group). The two groups of participants were matched for gender (nine female and four male), handedness (ten right, two left, one ambidextrous), and race (ten Caucasian, three Black African American). None of the participants had a history of psychiatric illness, organic brain disease, unstable medical illness, or history of serious head injury. The mean age for the chronic pain group was 51.8 years (SE 1.89, range 43 to 65) and the normal group was 48.7 years (SE 2.37, range 38 to 62 years). There was no significant difference in age between the two groups (T = 0.95, p = 0.36).

All chronic pain subjects suffered from muscle-skeletal low back pain. The diagnosis of muscle-skeletal low back chronic pain by the participant’s physician was based on several different criteria including: the duration of the participant’s complaint (minimum of six months), radiological or MRI type exams, tests performed for allodynia and hyperalgesia, confirmation of muscle atrophy, physical weakness, numbness or altered feeling, sleep disorders, memory dysfunction, and range of motion limitation. Additionally, physicians may have augmented their analysis with subjective participant-reported testing information such as the Pain Catastrophising Scale [37]; Waddell Signs [38], Visual Analog Scale [39], and the Fear Avoidance Belief’s Questionnaire [40] for example. None of the subjects in the normal control group reported suffering from chronic pain.

### Equipment

The equipment used in this experiment consisted of the following:

1. Seimens 1.5 Tesla Entera MRI scanner.
2. Food and Drug Administration (FDA) approved constant current stimulation device and pulse generation device.
3. Stimulating Electrodes.
4. Coaxial Shielded Lead.

Item one was used to gather and record data, and items two through four were used to time and deliver stimulation. The stimulation equipment used is compatible for use in the MRI scanner.

### Procedure

Participants filled out a questionnaire, were interviewed, and then questioned prior to being included in the study. To prepare for the experiment, the chronic pain participants were required not to take any medication for 12 hours prior to scanning. On the day of the experiment, the level of stimulation that each participant could withstand was determined by applying two self-adhesive electrodes to the participant’s lower lumbar area, one on each side of the spine. The electrodes were then connected to the leads of the constant current generator and pulse timing apparatus. A current of 0.4 mA was initially applied to the participant and increased in 0.1 mA increments until the participant indicated that this was the highest pain level that they could withstand for the duration of the study. This level, for each participant, was the level used during the fMRI experiment for that participant. The stimulation level for the chronic pain group (mean = 0.819 mA, SE = 0.116) and the normal group (mean = 0.662 mA, SE = 0.063) was not significantly different (T = 1.22, p = 0.25). The participants were instructed to remain still throughout the fMRI scanning procedure. Subjects were instructed to inform the experimenter if the pain level was too high and that the experiment could be discontinued at any time.

### Experiment

The experiment consisted of two conditions: electrical stimulation alternated with no stimulation (rest). Electrical stimulation while the participant lay in the scanner was conducted using the same equipment and level as had been previously determined for each individual. The electrical stimulation was delivered first for 14 seconds then followed by 14 seconds of no stimulation (rest). This sequence was repeated 5 times. The experiment consisted of two fMRI data gathering sessions (approximately 5.25 minutes each) separated by one session in which a T1 high-resolution (0.5×0.5×1 mm) anatomical brain scan was acquired (approximately 4.5 minutes) for each participant. The mean normalized anatomical T1 MRI scans (axial slices from Montreal Neurological Institute (MNI) z −50 to +85 in 3 mm steps) for the chronic pain group and the normal control group, are displayed in Figure S1. Total time for each participant in the MRI room was less than 15 minutes. The order in which subjects underwent scanning was random.

### fMRI Data Collection and Preprocessing

The experiment was conducted using a Seimens 1.5 Tesla Entera scanner. Functional T2* weighted images were acquired using a gradient echo-planar imaging sequence (repetition time 3670 ms, time to echo = 60 ms, flip angle = 90 degrees). A total of 36 interleaved axial slices were acquired with a 3×3×3 mm voxel resolution covering the cortex. A single run consisted of 86 scans (approximately 5.25 minutes). Images were preprocessed using SPM8 (Statistical Parametric Mapping version 8: Wellcome Department of Cognitive Neurology, University College London). Echo planar images (EPI) were unwarped and realigned. The images were then spatially normalized to MNI space (2×2×2 mm voxels) using a template T1 image and the mean EPI image as the source. The images were smoothed using an 8×8×8 mm FWHM Gaussian kernel.

Regional brain activity was assessed using a general linear model employing a boxcar function convolved with a hemodynamic response function (block design experiment). High pass filtering (cutoff period 128 seconds) was carried out to reduce the effects of extraneous variables (scanner drift, low frequency noise, etc). Auto-regression was used to correct for serial correlations. For each participant the contrast image of the electrical stimulation condition relative to the no stimulation condition was determined. We hypothesized that this contrasted image would differ for the participant with chronic pain compared to those without chronic pain.

### Feature Selection for the SLR Decoder

One major challenge for analyzing fMRI data, is the problem of over-fitting the data (an inability to generalize classification to novel test data), since there are far more features than subjects. We utilize two methods to reduce the number of features to overcome problems associated with over-fitting.

The first method was to utilize a multivariate pattern analysis technique, sparse logistic regression (SLR) [36]. SLR automatically selects only a few relevant features to be used for training and classification. This method has been shown to be quite effective for fMRI data where there are many more features than there are samples for training [36]. SLR is a Bayesian extension of logistic regression in which feature selection and training of the model parameters is performed simultaneously, selecting a few highly relevant features to be used for classification. This avoids problems related to over-fitting and enhances classification performance on novel data [36]. SLR utilizes supervised learning to train a classifier (SLR decoder) based on a known set of input stimuli. For a review of multivariate techniques applied to fMRI data see [36], [41]–[46].

The second method is to reduce the initial number of features by focusing only on brain regions known to be involved in pain processing, specifically chronic pain processing. We carried out our analyses only on brain regions found to be active during induced pain by Giesecke et al., [3]. The brain regions active during induced pain in both chronic pain subjects and normal control subjects (without chronic pain) were:

1. primary somatosensory cortex,
2. secondary somatosensory cortex,
3. inferior parietal cortex,
4. insula, and
5. anterior cingulate cortex.

While the cerebellum was included in the Giesecke et al., [3] study, it was not used in this study, since it wasn’t consistently included in the field of view in the fMRI scanning for all the subjects.

A mask was created using a standard MRI based anatomical atlas (WFU PickAtlas SPM toolbox). The mask (6686 voxels) of these regions of the brain (relative to 153595 voxels over the entire brain) was used for training and testing the SLR decoder.

### Training and Testing the SLR Decoder

Classification of each individual’s data was based on a statistical contrast image from each subject (the statistical parametric mapping (SPM) result of the electrical stimulation relative to no stimulation contrast image SPM con_0001.img) consisting of the masked voxels as described above. A leave-one-out cross validation technique was used to train and test the classification performance of the SLR. Using this cross-validation technique, 25 participants were used to train a SLR decoder which was then tested on the one sample that was left out. Independent SLR classifiers are trained using this procedure until all 26 samples have been tested in an unbiased manner. Performance was assessed by calculating the percent-correct for each of the two groups.

### Statistical Analyses

Non-parametric methods were used to assess the significance of the performance of the SLR decoder using permutation testing of 1000 randomly shuffled labels [13], [41]. This was accomplished by determining the number of times the performance of the decoder trained with accurate labeling was greater than the distribution of decoders trained with random labeling (assessed using p<0.05). Mean posterior accuracy and the posterior probability interval (serving as a confidence interval at p<0.05) were calculated based on procedures reported in [47]. Statistical significance of the selected features (using the false discovery rate (FDR) correcting for multiple comparisons pFDR <0.05, [48]) was determined in the following manner: The number of cross-validation iterations, out of 26, a feature (voxel) is selected by the SLR decoder using the real labels is compared to the distribution of the number of cross-validation iterations a feature is selected using 1000 permutations of randomly shuffled labels. Essentially, a comparison is made between the number of times a specific voxel is selected using the correct labeling relative to chance (random labeling).

Standard random-effects analyses was used to assess differences in brain activity between the two groups (using SPM8). A between subjects t-test was used to determine differences between chronic pain and normal control subjects over the masked voxels used to train the SLR decoders (using the stimulation relative to rest contrast images). Random-effects between subject t-tests, weighting the brain activity by pain threshold level, were conducted to determine whether differences in general properties of stimulation are responsible for the SLR decoding performance obtained in our study. An additional random-effects analysis was carried out for the chronic pain group to assess the correlation between brain activity and the duration since the onset of the chronic condition.

For the demographic variables of age and pain threshold level, Kolmogorov-Smirnov goodness-of-fit tests were conducted to determine if the data was normally distributed. The Wilcoxon rank sum test was used to test differences in the medians of the chronic pain group and the normal group for the variables of interest when the data was found not to be normally distributed.

The same preprocessing and statistical parametric analysis methods were carried out for all subjects in both the chronic pain group and the normal control group. The chronic pain versus normal group labeling that each subject belonged to was unknown to the person conducting these analyses. Given that the procedure is completely automated the programs do not have information regarding the group that a particular subject belongs to. This is not the case when training the multivariate pattern analysis classifiers. The utilization of supervised machine learning techniques, in this case sparse logistic regression, to train a classifier to distinguish between two groups requires that the labels be known. During testing, the classifier computes the predicted label of the individual based on the feature weights learned during training. It is important to emphasize that the classifier is completely blind to the true labels of the samples during testing.

## Results

The results of the SLR classification for the chronic pain and normal group are given in Table 1. The percent correct was determined by mean performance of the 26 classifiers trained using the leave-one-out cross-validation method explained previously. Significance at p<0.05 was determined using permutation tests of SLR classification over the randomly shuffled labels (1000 random samples) of the subjects in the training set. The overall classification accuracy for both groups together was 92.3% (p<0.05) with a D Prime of 2.924. The percent correct classification for the chronic pain group (sensitivity) was 92.3% (p<0.05) and for the normal group (specificity) was also 92.3% (p<0.05). Posterior mean accuracy and posterior probability intervals were computed using methods reported in [47] and given in Table 1.

**Table 1:** Performance of the Sparse Logistic Regression Classifier Chronic Pain versus Normal Group.

Mask	Overall Accuracy (%Correct)	Chronic Pain (%Correct) Sensitivity	Normal (%Correct) Specificity	Positive Predictive Value	Negative Predictive Value	Posterior Mean Accuracy	Posterior Probability Interval	D Prime
Pain Regions	92.3*	92.3*	92.3*	92.3	92.3	89.2	75.7–97.7	2.924

*Significant at p<0.05 utilizing 1000 permutation tests of sparse logistic regression SLR classification over randomly shuffled labels of the subjects in the training set. Percent correct was determined by the mean performance of the 26 classifiers trained using the leave one out cross validation method. Measures of sensitivity, specificity, positive predictive value, negative predictive value, posterior mean accuracy, posterior probability interval (p<0.05) and D Prime are also given. Posterior mean accuracy and posterior probability intervals were computed using methods given in Brodersen et al. (2010). The mask of voxels included in the analysis consisted of brain regions composing pain related areas: Primary Somatosensory Cortex, Secondary Somatosensory Cortex, Inferior Parietal Cortex, Insula, and Anterior Cingulate Cortex. The mask consisted of 6686 voxels. After training, 22 of the classifiers selected 3 features and 4 or the classifiers selected 2 features. The mean number of extracted features was 2.85.

The initial number of selected features before training was 6686. This was reduced by using the automatic relevance determination (ARD) characteristic of SLR [36], [49], [50] to a mean number of 2.85 extracted features across the 26 independently trained SLR classifiers (22 of the classifiers selected 3 features and 4 of the classifiers selected 2 features). It should be noted that the features are the individual voxels selected by the SLR classifier and the weights are the values assigned to each feature used for sorting the data into the different categories. Only five unique features (voxels) were used across the 26 independently trained SLR classifiers. The brain region of these selected voxels and their MNI coordinates are given in Table 2.

**Table 2:** MNI Coordinates for Sparse Logistic Regression Selected Voxels Weights Classifying Chronic Pain and Normal Individuals.

Brain Region of Selected Voxels	Positive Weights MNI x,y,z Coordinate	Negative Weights MNI x,y,z Coordinate	Number of Cross-Validation Iterations the Voxel was Selected	Number of Cross-Validation Iterations Correctly Classified	Number of Cross-Validation Iterations Incorrectly Classified
L S1 BA3		−42,−25,58	25*	23	2
L S1 BA3		−42,−22,58	1	1	0
L S1 BA3		−18,−43,61	25*	23	2
L IPC BA40	−57,−49,25		14*	14	0
L IPC BA40	−60,−49,25		9*	9	0

L = Left; BA = Brodmann Area; S1 = Primary Somatosensory Cortex; IPC = Inferior Parietal Cortex.

*Denotes significance at p<0.05 correcting for multiple comparisons of the number of cross-validation iterations out of 26 a voxel is selected by the sparse logistic regression SLR relative to the distribution of the maximum time a voxel is select by SLR over 1000 permutations of randomly shuffled labels of the subjects in the training set. Note that the five selected weights form three separate clusters of brain regions. The clusters composed of two weights consist of neighboring voxels.

**Figure 1:**
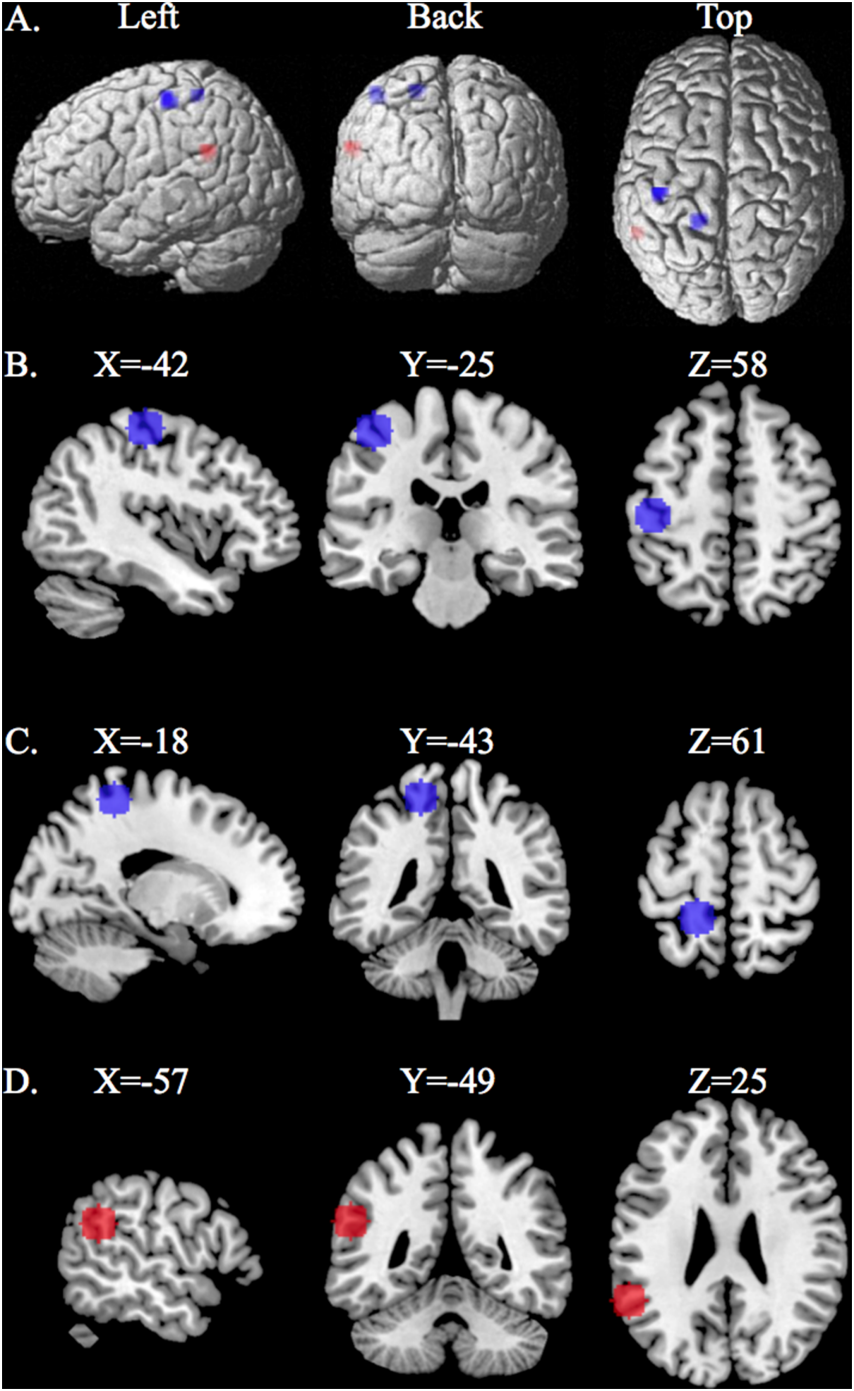
The three brain regions defined by the voxels selected by the sparse logistic regression located in the primary somatosensory cortex Brodmann Area BA 3 (MNI coordinates −42, −25,58 and −18,−43,61) consisting of negative weights (normal group greater than chronic pain group) and the inferior parietal cortex BA 40 (MNI coordinates −57,−49,25) consisting of positive weights (chronic pain group greater than normal group). A. Shows the three regions rendered on the surface of the brain. These three regions B. Somatosensory (MNI −42,−25,58), C. Somatosensory (MNI −18,−43,61), D. Inferior parietal cortex (MNI −57,−49,25) rendered on surface of MRI images sagittal, coronal, axial slices with MNI coordinates with an 8 mm sphere from center coordinate.

The five features (voxels) selected defined three clusters of brain activity: Two of the clusters were located in the left primary somatosensory cortex S1, and one of the clusters was located in the left inferior parietal cortex (Figure 1, Table 2). The features located in the primary somatosensory cortex S1 had negative weights (Figure 1 blue clusters) signifying that the chronic pain group had lower activity relative to the normal group. The features located in the inferior parietal cortex had positive weights (Figure 1 red cluster) signifying that the chronic pain group had greater activity relative to the normal group.

All three clusters had features that were significant correcting for multiple comparisons (Table 2). The only feature (voxel) that was not significant, only being selected for one SLR classifier, was adjacent to the feature (voxel) that was selected for the other 25 SLR classifiers.

Twenty-three of the correctly identified subjects had negatively weighted features from the primary somatosensory cortex S1 and positively weighted features from the inferior parietal cortex (IPC). The two subjects that were incorrectly classified (one chronic pain subject and one normal subject) involved features located in the primary somatosensory cortex S1.

A standard random-effects (SPM8) between-groups two-sample t-test was carried out using the same contrast images that were used during training and testing of the SLR decoder. There were no significant differences (positive or negative) between the chronic pain group (N = 13) and the normal control group (N = 13) using a corrected false discovery rate threshold of pFDR = 0.05. One cluster of activity in the inferior parietal cortex was found for the chronic pain over the normal control group within the pain related regions of interest using a lenient threshold of p<0.005 (MNI −60,−46,28; T = 2.88). For the normal control group over the chronic pain group four clusters of activity were found bilaterally in different parts of the postcentral gyrus in the somatosensory cortex with a lenient threshold of p<0.005 (MNI  = −48,−22,58, T = 4.79; −21,−46,64, T = 4.19; 60,−19,49, T = 3.44; 39,−28,55, T = 3.00).

To determine whether differences in general properties of stimulation were responsible for the SLR decoding performance obtained in the study, random-effects between-subject t-tests were conducted in which the brain activity (of the contrast images of stimulation relative to rest) was weighted by each individual’s pain threshold level. No significant difference in brain activity (positive or negative), between the chronic pain group (N = 13) and the normal control group (N = 13), was indicated using a corrected false discovery rate threshold of pFDR = 0.05 over the same voxels used to train the SLR decoder. The voxels selected by the SLR decoder (see Table 2) did not show any significant differential activity (p>0.05 uncorrected). Nor did a small-volume correction region of interest analysis (with an 8 mm search radius) detect any significant differential activity (p>0.05 corrected) around the voxels selected by the SLR decoder (see Table 2).

To assess the correlation between brain activity and the duration since the onset of the chronic condition, a random-effects analysis was conducted for the chronic pain participants in which the contrast images of stimulation relative to rest were weighted by the duration since the onset of the suffering of chronic pain. No significant correlation between brain activity (positive or negative) and duration since the onset of the suffering of chronic pain was indicated using a corrected false discovery rate threshold of pFDR = 0.05 over the same voxels used to train the SLR decoder. The voxels selected by the SLR decoder (see Table 2) did not show any significant differential activity (p>0.05 uncorrected), nor did a small-volume correction region of interest analysis (with an 8 mm search radius) detect any significant differential activity (p>0.05 corrected) around the voxels selected by the SLR decoder (see Table 2).

**Figure 2:**
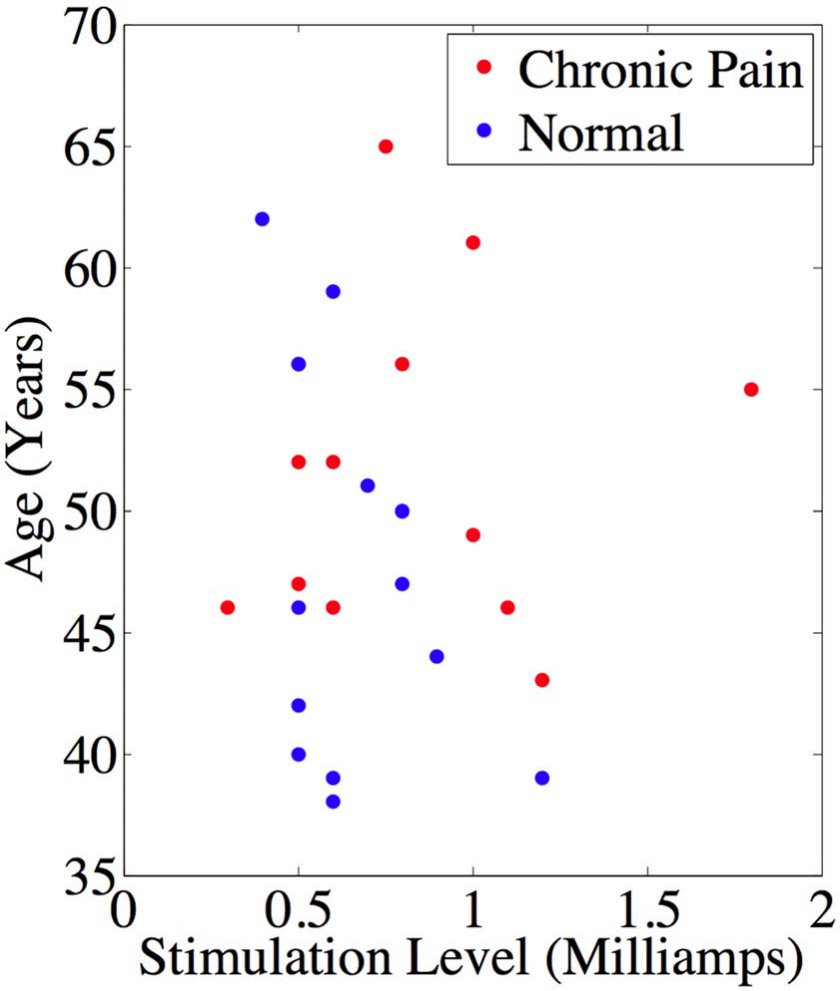
Plot of age (years) by pain threshold stimulation level (Milliamps) for each of the chronic pain (red) and normal control (blue) subjects.

The demographics of the chronic pain and normal control subjects are given in Table 3. As indicated, handedness, gender, and ethnicity are balanced across the two groups. Kolmogorov-Smirnov goodness-of-fit tests, over the variables of age and pain threshold level for each of the groups, indicated that the data was not normally distributed. The nonparametric Wilcoxon rank sum test was used to assess statistical differences (p<0.05 two-tailed) between the chronic pain group and the normal group for age (Median Chronic Pain Group = 52 years; Median Normal Group = 46 years; p>0.1 not significant) and pain threshold level (Median Chronic Pain Group = 0.75 mA; Median Normal Group = 0.6 mA; p>0.1 not significant) (See Figure 2). There was no correlation between age and stimulation level for either group (Pearson Correlation p>0.1). Neither was there a correlation between the duration of the chronic pain and pain threshold level or age (Pearson Correlation p>0.1).

**Table 3:** Demographics for Subjects.

Sub ID	Age	Sex	Hand	Ethnicity	Pain Thresh Level	Duration Since Onset of Condition	Meds Taken	Duration Meds Taken (Years)
*CP01	56	F	R	C	0.8	0.5	Diovan	0.167
							Ovar	2
							Marapex	3
							Ibuprofen	10
							Protonex	10
CP02	65	F	A	C	0.75	0.5	Vit Sup	0.5
CP03	52	F	R	C	0.6	5	Cambalta	2
							Motrin	2
CP04	61	F	R	C	1	2	None	0
CP05	55	M	L	C	1.8	1	Vit Sup	1
CP06	46	F	R	C	0.3	1	Dilantin	1
							Lavoxyl	1
							Vit Sup	1
CP07	47	F	R	C	0.5	2	None	0
CP08	43	M	R	C	1.2	5	Flexeril	15
							Skelaxin	0.67
CP09	46	M	R	B	1.1	0.5	Sevella	0.083
CP10	52	F	R	B	0.5	5	Ibuprofen	15
							Vit Sup	15
CP11	56	F	R	C	0.5	5	Percocet	5
							Mirapex	5
							Trazadone	5
CP12	49	M	L	C	1	5	None	0
CP13	46	F	R	B	0.6	0.5	Ibuprofen	8
NC01	39	F	R	C	1.2			
NC02	42	F	L	C	0.5			
NC03	62	M	A	C	0.4			
NC04	50	F	R	C	0.8			
NC05	59	F	R	C	0.6			
NC06	47	M	R	B	0.8			
NC07	46	F	R	B	0.5			
*NC08	51	F	R	C	0.7			
NC09	59	F	R	C	0.6			
NC10	44	F	R	C	0.9			
NC11	38	M	L	C	0.6			
NC12	40	F	R	C	0.5			
NC13	56	M	R	B	0.5			

Sex: F = Female; M = Male. Hand: L = Left; R = Right; a = Ambidextrous. Ethnicity: C = Caucasian; B = Black African American. Pain Thresh Level = Pain Threshold Level of electrical stimulation used in fMRI; Meds = Medication; Vit Sup = Vitamin Supplements.

*Denotes the two individuals misclassified by the Sparse Logistic Regression decoder. All Chronic Pain subjects were diagnosed with muscle-skeletal lower back chronic pain.

A Wilcoxon rank sum test was used to assess the statistical difference (p<0.05 two-tailed) in pain threshold between male (n = 8) and female (n = 18) participants (Median Male Group = 0.9 mA; Median Female Group = 0.6 mA; p>0.1 not significant). Even though our results show a tendency in the direction of higher thresholds for males it was not statistically significant. Although the results of several studies have shown that males have higher thresholds to experimentally induced pain than females (see [51] for review) there have been several studies that do not find statistical differences between males and females [51], [52].

Even though the chronic pain subjects had considerable variability in the duration from the onset of the chronic pain condition, the type of medication taken, and the duration the medication was taken, the SLR decoder was able to classify between the chronic and normal individuals with relatively high (92.3%) success. The two individuals that were misclassified by the decoder (one chronic pain and one normal control) do not appear to be outliers with regards to the demographic variables (see Tables 1 and 3).

To ensure the SLR classification results are not due to artifacts caused by differential head movement between chronic pain and normal subjects, between group analyses were conducted using the SPM realignment parameters. The summed scan-to-scan difference in the realignment parameters (tested separately for the six realignment parameters: Translation X, Y, Z dimensions, and rotation pitch, roll, yaw dimensions) did not show any statistically significant differences between the chronic pain and normal groups (See Table S1). Analysis of the total deviation within a session of the six realignment parameters also revealed no statistically significant difference between the chronic pain and the normal group (See Table S2).

A post-hoc calculation of power was conducted (using procedures given in [53]) to determine the sensitivity of the results with the sample size used in our study. Given the incidence of a positive classification in the chronic pain group of 0.923 with a standard deviation of 0.277, and the incidence of a positive classification in the normal group of 0.077 with a standard deviation of 0.277 using a two-sided 95% confidence interval, the power is 100% confirming the sensitivity of the results given the sample size for each group. Power values of 80% are considered to be standard in sample size calculations [53]. The posterior probability interval [47] (given in Table 2) similar to a confidence interval indicates the reliability at estimating the unknown population parameter given a two-tailed 95% confidence level. The posterior probability interval in our study using 13 subjects per group ranges from 75.7% to 97.7%, which is well above the chance level of 50%. Increasing the number of subjects will decrease the posterior probability interval and increase the reliability of the classification results.

## Discussion

The results of the experiment show that multivariate pattern analysis (specifically SLR) of the fMRI contrast image of induced pain relative to rest can be used to classify individuals with chronic pain with a mean accuracy of 92.3% (Table 1). This classification performance reflects that of novel test samples of a single contrast image (not included in the training), using a leave-one-out cross-validation technique. This degree of classification accuracy is quite impressive when one considers that no statistically significant differential activity correcting for multiple comparisons is present between the chronic pain group and the normal control group using traditional univariate random-effects analysis over the same data. Similarly, it is interesting to point out that the study by [3], [54] did not find consistent differences between chronic pain and normal groups for acute pain stimulation using univariate analyses. The high predictive performance of the multivariate decoder and the lack of strong univariate differential activity, strongly suggests that (multivoxel) patterns of activity are neurological markers that can be utilized for identifying individuals with chronic pain.

The SLR decoder selected features in the left inferior parietal cortex and features from two different regions along the postcentral gyrus in the primary somatosensory cortex (Figure 1, Table 2). These regions are part of the so called ‘pain matrix’ [3], [5], [55], [56]. This region of the inferior parietal cortex (MNI coordinates −57, −49, 25) has been associated with heat stimulation [57] and cold pain [58]. One region of the somatosensory cortex (MNI coordinates −18,−43,61) is consistent with somatotopic representation of the trunk region [59], [60], whereas, the other (MNI coordinates −42, −25, 58) is consistent with somatotopic representation of the hand [59], [60]. It is interesting to point out that individual decoders trained on voxels from the primary somatosensory cortex were able to significantly classify near-threshold pain versus no-pain in the study conducted by [24] but were not able to significantly classify between painful and nonpainful stimuli in normal individuals in the Brown et al., [13] study.

The use of several different brain regions by the SLR decoder is consistent with the view of pain being a distributed process [9], [24], [61]. While studies using a multivariate pattern analysis have shown that classification of pain versus no pain significantly utilizes activity in the insula [9], [13] and anterior cingulate [9] these regions did not contribute to classifying individuals with chronic pain from normal individuals based on our results. These results suggest that while these regions are important for distinguishing painful from non-painful stimuli the processes in these regions may not differ between individuals with chronic pain and normal individuals. Another possibility may be that the activity in these regions is similar to activity in the somatosensory and inferior parietal regions selected by the SLR decoder. One characteristic of SLR is that it will eliminate features that are similar in order to improve generalization performance. This is perhaps one reason why features are not bilaterally represented along the somatosensory strip as one would expect given that electrical stimulation included both left and right sides of the back. With a larger training sample size it is likely that more features will be selected while maintaining high generalization performance.

The goal of this study was not to classify between pain and non-painful stimuli as has been done in many other studies [9], [13], [21]–[26], but rather to classify individuals with chronic pain from normal individuals by the patterns of the contrast in activity recorded between painful stimulation relative to rest. It has been conjectured that individuals with chronic pain may have a reorganization of processes within brain regions that is responsible for their ongoing sensation of pain [3], [29]–[31]. In the study conducted by Ung et al., [35], multivariate pattern analysis over anatomical MRI of gray matter density was used to successfully classify individuals with chronic pain from normal controls with a accuracy of 76%. The weights of the support vector machine responsible for distinguishing the two groups were consistent with grey matter density decreases in chronic pain individuals in the right borderline amygdala, left medial orbital gyrus, and right cuneus; as well as grey matter density increases in the right cerebellum, regions of the temporal lobe, left primary and secondary somatosensory cortices, left primary motor cortex, the right calcarine sulcus, and the right dorsolateral prefrontal cortex. Given the increased grey matter density in somatosensory cortex in the Ung et al. [35] study one may expect that in our study, individuals with chronic pain would show greater activity in these pain related brain regions under equal ‘subjective’ pain conditions than normal healthy individuals. (However, see [30], [31], [33] in which grey matter atrophy was found in individuals with chronic pain in many brain regions including the somatosensory cortex). While greater activity was found in the chronic pain group in the inferior parietal cortex (signified by positive weights meaning chronic pain > healthy normal group), it was not the case for activity in the primary somatosensory cortex (signified by negative weights meaning chronic pain < healthy normal group). Our results are more in line with studies [30], [31], [33] showing grey matter atrophy in somatosensory cortex as a possible reason for less activity being shown in response to electrical stimulation. While one may predict greater neural reorganization with the duration of chronic pain (denoted by changes in brain activity), this was not indicated by the random-effects analysis of the correlation between duration since onset of the suffering of chronic pain with that of brain activity (see Results). It may be the case that reorganization of neural processing mostly occurs within the first six months from the onset of suffering and does not show a linear relationship with time. In any case, the reorganization in brain activity that takes place was differentiated from that of normal controls by the SLR decoder even with only six months since the onset of suffering chronic pain for some individuals.

There are several potential reasons why the SLR classifier weighted activity in the somatosensory cortex in the direction of normal individuals. Besides possible grey matter atrophy [30], [31], [33], another possibility may be that individuals with chronic pain have a reorganization in inhibitory networks used to suppress pain that causes a reduction in somatosensory cortex activity under intense pain. There is some evidence suggesting potential motor inhibition during painful stimulation in chronic pain individuals [62]. It has been shown in rats that induced motor cortex stimulation can alleviate chronic pain and has the effect of suppressing somatosensory evoked potentials [63]. While there are studies reporting increased activity in somatosensory cortex to painful stimulation for individuals with chronic pain relative to normal individuals [29], it is possible that in the high pain condition in our study, the individuals with chronic pain may be using acquired pain coping strategies that suppress activity within the somatosensory cortex. Indeed, there have been several studies in which higher pain thresholds are found for individuals with chronic back pain [64], [65].

It is also possible that chronic pain individuals have some degree of residual pain when stimulated that extends longer than healthy normal individuals. Indeed it has been conjectured that chronic pain may resemble that of a persistent memory trace that cannot be extinguished [5], [31]. It may also be the case that the somatosensory cortex always has some degree of activation in chronic pain individuals associated with their chronic sensation of pain in the absence of any stimulation. This heightened response in the resting state may also result in less differential activity for the chronic pain individuals relative to healthy normal individuals. There is some evidence that the resting state networks of individuals with chronic pain may be different from that of normal healthy controls [31], [54], [66]. Several aspects of this differential activity need clarification in further research.

It is important to ensure that the classification performance of a decoder is the result of the features under investigation and not due to some extraneous confound that may be present between the chronic pain group and normal groups. One potential confound that could exist between the two groups is greater head movement in the MRI scanner for individuals with chronic pain. However, statistical analysis of the six realignment parameters did not reveal any differences between the chronic pain and normal control groups (See Tables S1 and S2).

An additional confound that one may expect under equal pain conditions is differing levels of stimulation between the two groups. Some studies have shown that pain sensitivity is a correlate of chronic pain status [10]. When controlling for age, gender, and ethnicity, we did not find any significant difference in the mean threshold level of electrical stimulation between the chronic pain and the normal control groups. Finding no difference between the two groups is desirable, the results of the SLR decoder cannot simply be explained by differential activation in brain regions resulting from different stimulation levels. Indeed the analysis in which the brain activity was weighted by each individual’s pain threshold level, showed no significant difference between the chronic pain group and the normal control group. While some studies have shown lower thresholds for pain in individuals suffering from chronic low back pain [3], [10] this is not always the case [29], [64], [65]. In the study conducted by [29] no difference in electrical stimulation pain thresholds were detected between individuals with chronic low back pain and normal controls. It has also been shown in some studies that individuals with chronic low back pain have higher heat pain thresholds than normal controls [64], [65]. Given the individual variability and the inconsistency in the published literature on the direction and significance of the difference between individuals with chronic low back pain and normal controls it is unlikely that pain threshold level will serve as a good diagnostic measure above that of the fMRI based SLR decoder presented here.

It has been shown that there are differences in the pain associated neural activity using different modalities of stimulation [67]. There are also differences in the temporal characteristics of the onset of the perception of pain with electric being immediate and thermal slower. Because we used only one type of stimulation modality it is possible that the differences in brain activity used by the classifier to distinguish between chronic pain and normal individuals may be a result of brain activity to properties of stimulation that are separate from those of processing induced acute pain. However we do not believe this to be the case here for the following reasons: 1. There was no statistically significant difference in the pain threshold levels used for stimulation for the chronic pain and normal control group. 2. The between-subject analysis in which brain activity was weighted by pain threshold level was not significant in the voxels selected by the SLR decoder (small volume correction analyses using a radius of 8 mm were also not found to be significant) between the chronic pain group and the normal control group. Both of these findings make it unlikely that general properties of stimulation are responsible for the classification performance reported in our study. Rather the results are more consistent with differences between chronic pain and normal individuals in the processes related to the perception of induced acute pain. Even if is found that the ability to discriminate individuals with chronic pain from normal individuals does not generalize to other modalities of stimulation it would not change the relevance of the results of our classification performance using SLR to distinguish between chronic pain and normal individuals using electrical stimulation.

## Conclusions

The combination of the experimental design and the multivariate pattern analysis of fMRI data presented in this study allowed us to recognize brain markers for chronic pain. The experimental method of using contrast images of painful stimulation versus rest enabled us to successfully identify the presence of chronic pain. This provides further evidence that individuals with chronic pain process induced pain differently from normal individuals and that this difference may arise from reorganization in brain processes resulting from the pathology. This study employs the use of multivariate pattern analysis techniques, specifically sparse logistic regression SLR [36], to overcome problems of over-fitting and to successfully classify individuals with chronic pain from normal control individuals with 92.3% classification accuracy with high sensitivity and specificity (Table 1). While a small sample size was used, only 13 individuals in each group, the unbiased analysis methods performed in this study suggest that these results should be reliable and valid for classifying new individuals using this procedure. Future studies need to be conducted using larger group sizes with greater variability in population demographic variables such as race, gender, and age. With the use of a larger sample size the posterior probability interval will decrease around the mean posterior accuracy providing a more precise predictive measure.

Future studies should explore the effects that different forms of noxious stimulation (thermal, pressure, electric) and different levels of stimulation (equal physical level, equal just noticeable pain threshold, equal high pain threshold) have on classification performance. The general applicability of the classifier would be greatly enhanced if it were found that different types of stimulation could be used for testing even though the classifier is only trained on a specific type of stimulation being used. In the work conducted by [9] the classifier trained to detect the presence of acute pain using thermal stimulation was able to generalize to detect acute pain using an opiate analgesic. Future studies should determine whether the classifier generalizes across data collected on different MRI scanners.

In the present study only muscle-skeletal chronic low back pain was investigated. It would be interesting in future studies to determine if classifiers can generalize across different types of chronic pain, whether certain types of chronic pain are more easily classified, and whether performance of the classifiers can be enhanced by specifically training on each type of chronic pain.

This study presents a method by which chronic pain can be diagnosed from fMRI contrast images acquired in response to induced acute pain. The brain-based markers determined by multivariate pattern analysis can be used to objectively determine whether an individual has chronic pain. This could have great clinical significance in the diagnosis and treatment of individuals suffering from chronic pain.

## Supporting Information

Figure S1 **Mean T1 normalized anatomical MRI axial slices for the chronic pain group and the normal control group.** MNI z coordinates of −50 to +85 at 3 mm intervals. Left side of image is left side of brain.
(TIFF)

Table S1 **Summed Scan-to-Scan Difference in Realignment Parameters: Image Translation: X, Y, Z Dimensions and Rotation: Pitch, Roll, Yaw dimensions.**
(DOCX)

Table S2 **Total Deviation of the Six Realignment Parameters: Image Translation: X, Y, Z Dimensions and Rotation: Pitch, Roll, Yaw dimensions.**
(DOCX)
