# Supplementary figures and images for: A Tool for Classifying Individuals with Chronic Back Pain: Using Multivariate Pattern Analysis with Functional Magnetic Resonance Imaging Data
Source: PLoS One. 2014 Jun 6;9(6):e98007. doi: 10.1371/journal.pone.0098007 (PMC4048172; doi:10.1371/journal.pone.0098007)

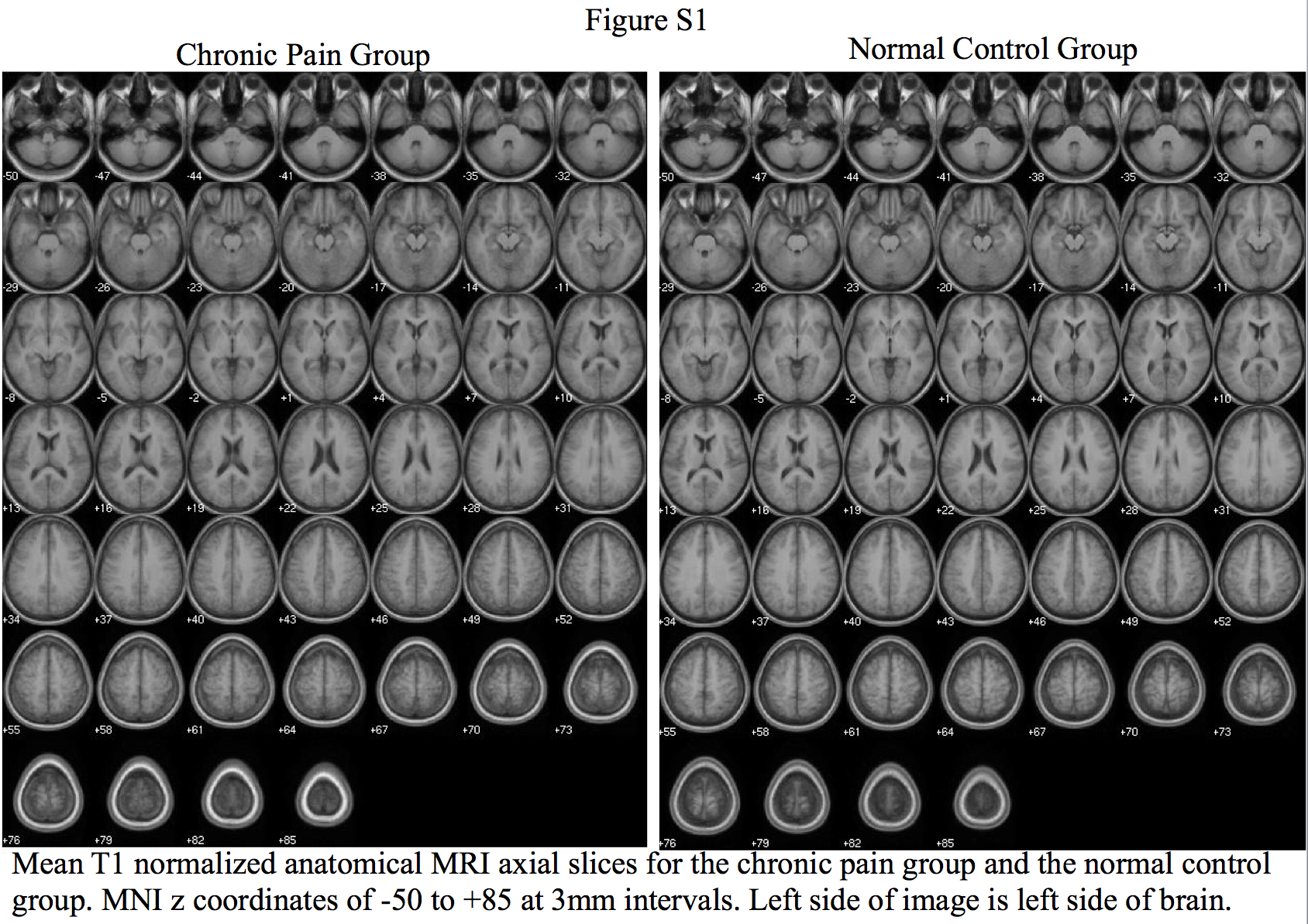

Supplement: Figure S1 — Mean T1 normalized anatomical MRI axial slices for the chronic pain group and the normal control group. MNI z coordinates of −50 to +85 at 3 mm intervals. Left side of image is left side of brain. (TIFF) [file pone.0098007.s001.tiff]
