# Supplementary material for: A Tool for Classifying Individuals with Chronic Back Pain: Using Multivariate Pattern Analysis with Functional Magnetic Resonance Imaging Data
Source: PLoS One. 2014 Jun 6;9(6):e98007. doi: 10.1371/journal.pone.0098007 (PMC4048172; doi:10.1371/journal.pone.0098007)
Supplement: Table S1 — Summed Scan-to-Scan Difference in Realignment Parameters: Image Translation: X, Y, Z Dimensions and Rotation: Pitch, Roll, Yaw dimensions. (DOCX) [file pone.0098007.s002.docx]

Table S1

Summed Scan-to-Scan Difference in Realignment Parameters

Image Translation: X, Y, Z Dimensions and Rotation: Pitch, Roll, Yaw dimensions

|  |  |  | Chronic Pain Group | Chronic Pain Group | Normal Group | Normal Group |
| --- | --- | --- | --- | --- | --- | --- |
|  | t-tests | p-value | Mean | SE | Mean | SE |
| X | 0.09 | 0.92 | 1.99 mm | 0.29 | 2.05 mm | 0.47 |
| Y | -0.20 | 0.85 | 4.75 mm | 1.02 | 5.18 mm | 2.03 |
| Z | -0.56 | 0.58 | 6.17 mm | 1.33 | 8.18 mm | 3.46 |
| Pitch | -0.62 | 0.54 | 0.10 degrees | 0.02 | 0.14 degrees | 0.06 |
| Roll | -0.59 | 0.56 | 0.04 degrees | 0.005 | 0.05 degrees | 0.01 |
| Yaw | -0.28 | 0.78 | 0.04 degrees | 0.004 | 0.04 degrees | 0.01 |

No statistically significant differences were found between the chronic pain group and the normal (control) group.
