# Supplementary material for: A Tool for Classifying Individuals with Chronic Back Pain: Using Multivariate Pattern Analysis with Functional Magnetic Resonance Imaging Data
Source: PLoS One. 2014 Jun 6;9(6):e98007. doi: 10.1371/journal.pone.0098007 (PMC4048172; doi:10.1371/journal.pone.0098007)
Supplement: Table S2 — Total Deviation of the Six Realignment Parameters: Image Translation: X, Y, Z Dimensions and Rotation: Pitch, Roll, Yaw dimensions. (DOCX) [file pone.0098007.s003.docx]

Table S2

Total Deviation of the Six Realignment Parameters

Image Translation: X, Y, Z Dimensions and Rotation: Pitch, Roll, Yaw dimensions.

|  |  |  | Chronic Pain Group | Chronic Pain Group | Normal Group | Normal Group |
| --- | --- | --- | --- | --- | --- | --- |
|  | t-tests | p-value | Mean | SE | Mean | SE |
| X | 0.55 | 0.58 | 0.35 mm | 0.07 | 0.40 mm | 0.06 |
| Y | -0.14 | 0.89 | 0.70 mm | 0.08 | 0.73 mm | 0.17 |
| Z | -0.38 | 0.71 | 1.09 mm | 0.34 | 1.14 mm | 0.35 |
| Pitch | 0.38 | 0.71 | 0.017 degrees | 0.003 | 0.02 degrees | 0.008 |
| Roll | -0.17 | 0.87 | 0.008 degrees | 0.002 | 0.008 degrees | 0.001 |
| Yaw | -0.19 | 0.85 | 0.007 degrees | 0.002 | 0.008 degrees | 0.002 |

No statistically significant differences were found between the chronic pain group and the normal (control) group.
